# A Multi-Nutrient Stoichiometric Framework Reveals Distinct Plant–Soil Responses to 12 Years of Nitrogen Fertilization and Mowing in an Agro-Pastoral Ecotone Grassland

**DOI:** 10.3390/plants15142136

**Published:** 2026-07-10

**Authors:** Muqier Hasi, Canran Yang, Yasong Chen, Yibo Li, Jianhui Huang, Yinliu Wang, Guoxiang Niu

**Affiliations:** 1 College of Grassland Science, Inner Mongolia Agricultural University, Hohhot 010011, China; hasimuqier@imau.edu.cn; 2 Jiangxi Provincial Key Laboratory of Carbon Neutrality and Ecosystem Carbon Sink, Lushan Botanical Garden, Jiangxi Province and Chinese Academy of Sciences, Jiujiang 332900, China; yangcr@lsbg.cn (C.Y.); yasongchen@foxmail.com (Y.C.); 3 Key Laboratory of Land Surface Pattern and Simulation, Institute of Geographic Sciences and Natural Resources Research, Chinese Academy of Sciences, Beijing 100101, China; liyb.18b@igsnrr.ac.cn; 4 Key Laboratory of Vegetation and Environmental Change, Institute of Botany, Chinese Academy of Sciences, Xiangshan, Beijing 100093, China; jhhuang@ibcas.ac.cn; 5 Shandong Key Laboratory of Eco-Environmental Science for the Yellow River Delta, Shandong University of Aeronautics, Binzhou 256603, China; wangyinliu@ibcas.ac.cn; 6 Senckenberg Museum of Natural History Görlitz, 02826 Görlitz, Germany

**Keywords:** nutrient stoichiometry, fertilization, mowing, plant–litter–soil continuum

## Abstract

Nutrient stoichiometry provides a powerful framework for linking nutrient limitation to plant community biomass, especially in grasslands undergoing degradation in the agro-pastoral ecotone, where nitrogen (N) fertilization and mowing have become two widespread key management practices. However, their influence on nutrient stoichiometry has received little attention, especially beyond the leaf carbon (C):N:phosphorus (P) ratio. Here, we conducted a field experiment on the Mongolian Plateau wherein we quantified 19 nutrient ratios for soils and three plant components (aboveground plants, litter, and belowground roots) following 12 years of N addition (0, 2, and 10 g N m^−2^ year^−1^) combined with mowing, and grouped these ratios into four sets with C, N, P, and potassium (K) as the numerators. Under N addition, nutrient stoichiometry in plant components and soils changed markedly, whereas mowing management resulted in negligible changes in most C-, N-, and K-based nutrient ratios. Furthermore, mowing and N addition interactively and significantly affect P-based nutrient ratios. The responses of nutrient stoichiometry differed among plant components and soils, and also depended on the level of N input, and these ratios with C and N as numerators generally showed greater variability than those with P and K in the plant–soil system. Plant community biomass was associated with nutrient ratios in both plant components and in soils, although the relationships were not always significant. Long-term N addition resulted in rate-dependent shifts in nutrient stoichiometry, whereas mowing had only weak modifying effects. Extending nutrient stoichiometry framework (including neglected ratios, e.g., N:K) beyond leaf C:N:P to encompass entire plant–soil systems can help local government and ranch owners manage grasslands more cost-effectively because of simple assessment procedures, and could further provide more comprehensive insights into nutrient limitation and main hypotheses of ecological stoichiometry in grassland ecosystems.

## 1. Introduction

Grasslands occupy roughly 69% of global agricultural land and contribute approximately one-third of net primary production on Earth’s land surface [1,2]. The biomass and carbon storage of these systems underpin human well-being and help slow ongoing climate warming [3,4]. Throughout their life cycle, grasses require a suite of mineral nutrients. In addition to nitrogen (N) and phosphorus (P), which are the most frequently studied limiting elements, other mineral nutrients (e.g., calcium, Ca; magnesium, Mg; iron, Fe; manganese, Mn) can also restrict grassland productivity and functioning [5,6,7,8]. For example, a recent study showed that aboveground production in grassland ecosystems increased by more than 10% under potassium (K) fertilization in field trials, emphasizing the importance of K limitation in global terrestrial ecosystems [9].

Nutrient stoichiometry is widely regarded as a powerful tool for investigating nutrient limitation in grassland ecosystems [10,11]. Plant nutrient ratios are expected to remain relatively constrained, even under environmental change, and large shifts in these ratios may have serious negative consequences for plant growth [12]. Many previous studies have relied on threshold values of the leaf N:P ratio (i.e., 20:1 vs. 10:1 or 16:1 vs. 14:1) to infer nutrient limitation and have concluded that terrestrial production by both grasses and vascular plants is strongly regulated by the N:P ratio [13,14,15]. Nonetheless, some recent studies are challenging these points, and reported that leaf N:P ratio was ineffective in predicting plant nutrient limitation based on large-scale observed studies and long-term manipulation experiments [16,17,18]. A recent study addressed the importance of leaf P fractions, and further explained why leaf N:P ratio was not reliable for diagnosing plant nutrient limitation of productivity [18]. Other nutrient ratios can also provide insights into nutrient limitation. For example, plant N:K and P:K ratios have been used to detect K limitation in terrestrial ecosystems, although the specific thresholds vary among ecosystem types and regions [9,19,20]. Ratios of N:manganese (Mn) and P:Mn can serve as indicators of Mn toxicity in grass, and the K/(Ca + Mg) ratio indicates the potential risk of grass tetany for ruminants in grassland systems [9,19,20,21,22]. Most previous work has focused on nutrient stoichiometry in leaves [23]; our understanding of the dynamics of nutrient ratios in other plant organs and soils is limited.

Mowing and N fertilization have become widely used management practices in grasslands undergoing degradation worldwide [4,24,25]. Mowing removes aboveground biomass (AGB) in a non-selective manner, providing hay for large herbivores and reducing the dominance of species with low nutrient use efficiency, while favoring those with higher nutrient use efficiency [26]. In many restoration efforts targeting degraded grasslands under mowing regimes, N fertilization is often used as a complementary measure to enhance soil fertility and increase AGB [27]. Additional N inputs generally improve ecosystem N status and stimulate plant nutrient uptake, thereby altering nutrient stoichiometry by, for example, increasing N:P and reducing C:N value [28,29]. Furthermore, N addition can influence the concentrations of other nutrients (e.g., Ca, Mg, Fe) in plant–soil systems, through either concentration or dilution effects on these mineral elements [30,31]. For example, to maintain the relatively stable plant nutrient stoichiometry, the plant will adjust the distribution of Mn and P, and more Mn and less P will be stored in the roots and litter to avoid Mn poisoning and improve P utilization [32,33]. However, to date, an absence of multi-nutrient stoichiometric frameworks at the community scale and unclear relationships between plants and soils have substantially impeded the practical implementation and wider adoption of the theory in grassland management.

More than 70% of grasslands on the Mongolian Plateau have experienced some degree of degradation, and N fertilization, together with autumn mowing, are commonly applied management approaches in this region. In 2008, we initiated a field experiment to examine how these two practices influence ecosystem nutrient cycling [34,35,36]. Our aim was to assess the effects of long-term N addition and mowing on 19 nutrient ratios measured in three plant components and soils, and further explore nutrient limitations by analyzing relationships between plant and soil nutrient ratios. All 19 nutrient ratios were grouped into four classes with C, N, P, and K as the respective numerators. Here, we hypothesized that after 12 years of N addition and mowing management, N addition would exert stronger effects on N nutrient ratios than on other nutrient ratios in plants and soils, and that these effects would be further modulated by mowing. In addition, we further discussed the potential mechanisms driving variations in nutrient ratios in plant–soil systems based on the major existing hypotheses in plant ecological stoichiometry (e.g., Growth Rate Hypothesis), and we further proposed a preliminary theoretical framework to guide future research in grassland ecosystems.

## 2. Results and Discussions

**Figure 1:**
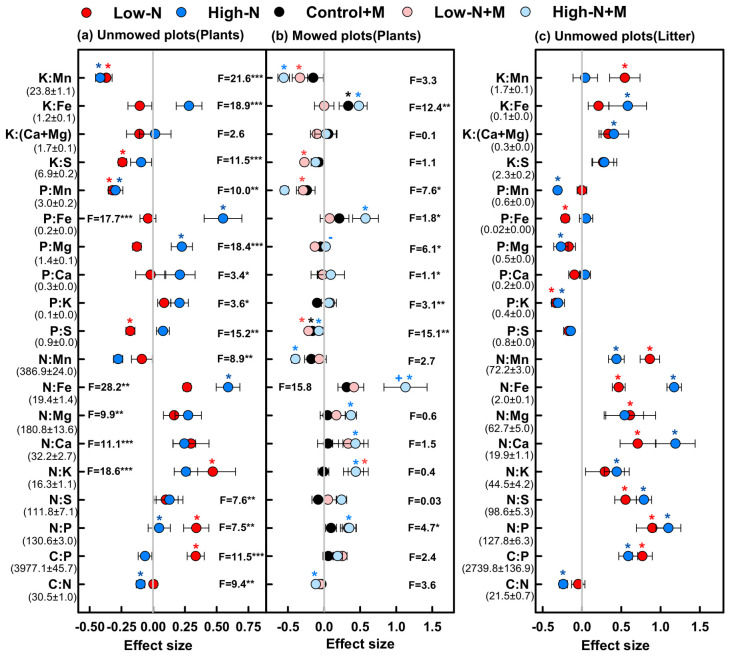
Effects of nitrogen (N) addition and mowing (M) on 19 nutrient stoichiometric ratios in aboveground plants and litter in an Inner Mongolian grassland. (**a**) Plants in unmowed plots; (**b**) plants in mowed plots; and (**c**) litter in unmowed plots. Notes: colored circles with error bars depict mean ± SE (n = 4). Mean nutrient ratios for the control plots are shown beneath each corresponding ratio. Effect sizes are expressed as deviations in nutrient ratios relative to the controls. Bars or points to the left of zero represent decreases, whereas those to the right of zero indicate increases. An asterisk (*) denotes a significant difference between N or M treatments and the control at *p* < 0.05. Two-way ANOVA results (F-values, ***, **, and * indicating significant effects at *p* < 0.001, 0.01, and 0.05, respectively) for the effects of N (**a**,**c**) and M on nutrient ratios are displayed at the appropriate positions for unmowed and mowed panels, with only significant cases labeled. The symbols “+” and “–” indicate increased and decreased effects of mowing as compared with unmown plots under the same N addition dose based on *t*-test results, respectively.

**Figure 2:**
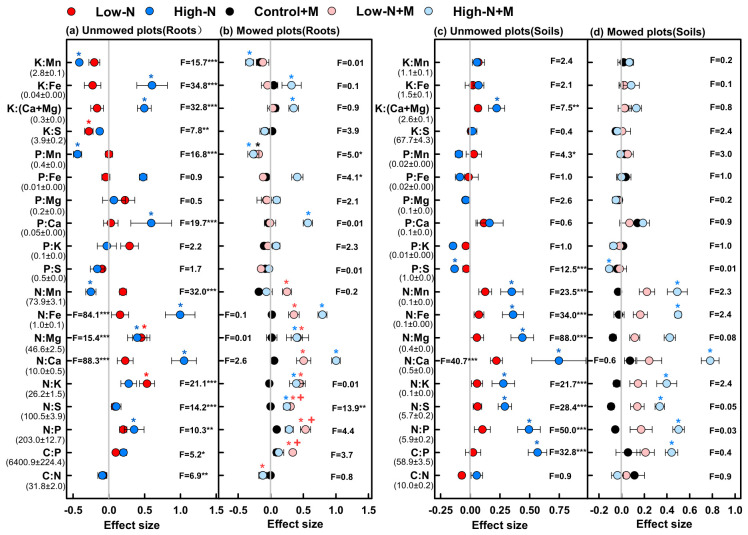
Effects of nitrogen (N) addition and mowing (M) management on the nineteen nutrient stoichiometric ratios in roots and soil in an Inner Mongolian grassland. (**a**) Roots in unmowed plots; (**b**) Roots in mowed plots; (**c**) Soils in unmowed plots; and (**d**) Soils in mowed plots. Notes: colored circles with error bars depict mean ± SE (n = 4). Mean nutrient ratios for the control plots are shown beneath each corresponding ratio. Effect sizes are expressed as deviations in nutrient ratios relative to the controls. Bars or points to the left of zero represent decreases, whereas those to the right of zero indicate increases. An asterisk (*) denotes a significant difference between N or M treatments and the control at *p* < 0.05. Two-way ANOVA results (F-values, ***, **, and * indicating significant effects at *p* < 0.001, 0.01, and 0.05, respectively) for the effects of N (**a**,**c**) and M on nutrient ratios are displayed at the appropriate positions for unmowed and mowed panels, with only significant cases labeled. The symbols “+” and “–” indicate increased and decreased effects of mowing as compared with unmown plots under the same N addition dose based on *t*-test results, respectively.

**Figure 3:**
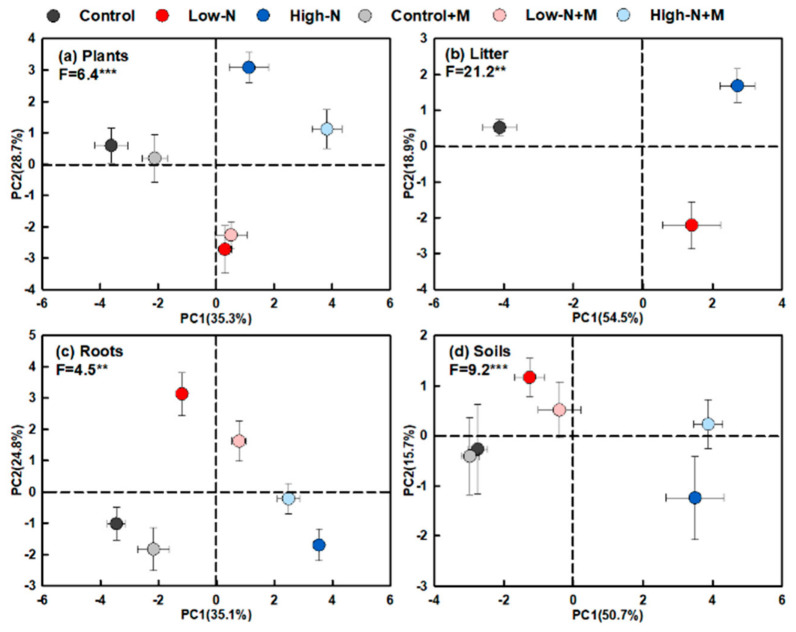
Overall shifts from the control to nitrogen (N) and mowing (M) treatments illustrated by principal component analysis (PCA). (**a**) Plants; (**b**) litter; (**c**) roots; and (**d**) soils. Nineteen nutrient ratios were used to quantify these responses, with all ratios for each treatment condensed into a single point in PCA. The PERMANOVA results are shown to indicate the significance. Notes: ***, and ** indicate significance at *p* < 0.001 and 0.01, respectively.

After 12 years of combined N addition and mowing, N addition markedly changed nutrient stoichiometry in all three plant components and in soils, whereas mowing alone had little effect on most C-, N-, and K-related ratios (Figure 1, Figure 2 and Figure 3). PCA and ANOVA revealed that N addition clearly had a stronger overall effect than mowing on the 19 nutrient ratios in the plant–soil system, and ratios with C and N as numerators varied more than P and K ratios (Figure 1, Figure 2 and Figure 3). Overall, these findings were consistent with our hypothesis. Previous studies have examined the joint effects of mowing and N fertilization on nutrient dynamics in grassland ecosystems and have emphasized the main effects of N fertilization; however, the regulatory role of mowing under elevated N (i.e., whether it constrains, amplifies, or weakens N effects) has been reported to vary among grassland types and with both the amount and duration of N addition [11,37,38,39].

In our experiment, we found that mowing had only a limited capacity to alleviate the influence of N addition on multi-nutrient stoichiometry. This pattern may arise for two main reasons. First, long-term N inputs can substantially alter plant community biomass, biomass allocation, and soil conditions (e.g., pH, soil acid neutralizing capacity), which in turn modify nutrient stoichiometry [27,40]. A recent study reported that the decadal increasing effects in typical grassland biomass production from chronic N additions could be eliminated by annul mowing, and highlighted the importance of dynamics of dominant species and community species richness [41]. Another study near our experiment site also found that mowing generally decreased soil N availability, while the concurrent decrease in soil moisture slowed nutrient acquisition and utilization, resulting in greater height C3 forbs than C3 grasses [42]. Second, in this study, a 10 cm layer of aboveground plant residues was retained after mowing, providing additional nutrient inputs through decomposition. Mowing generally removes macroelements (e.g., C, N, P, K) rather than microelements (e.g., Fe, Mn) via the biomass harvest method. Together, these mechanisms help explain why C and N ratios showed greater variability than P and K ratios. Ratios with C and N as numerators for the three plant components and soils were significantly associated with the PC1 axis, except for soil C:N (Table S1). To date, relatively few studies have directly evaluated how mowing stubble height or the frequency and time of mowing influences nutrient dynamics, and found that plant biomass declined while nutrient contents increased as mowing stubble height decreased [37,38,39,43].

Indeed, changes in community species composition (e.g., shifts in dominant species) and community phenotypic plasticity (e.g., biomass allocation and root traits), especially of dominant species, conjointly influence nutrient ratio dynamics in plant–soil systems irrespective of mowing and N addition [18,44]. On the one hand, extra N inputs can generally decrease most N-related nutrient ratios (N as the numerator, e.g., N:P) in the whole plant–soil system especially through increasing the biomass and richness of dominant species [26,45]. On the other hand, many studies reported that mowing could enhance community species richness and decrease the competitive advantage of dominant species under long-term N addition by AGB removal, increasing sub-canopy light intensity and promoting germination across multiple species [42,46,47,48]. Nonetheless, we also observed that mowing significantly affected K:Fe and N:P ratios, all P-based ratios (P as the numerator) in aboveground plants, and N:S, P:Fe, and P:Mn ratios in roots (Figure 1 and Figure 2). A recent study likewise showed that mowing can intensify plant P limitation and the regulation of P acquisition under N fertilization in a temperate meadow [37]. According to the Growth Rate Hypothesis, N addition will increase the plant growth rate and decrease plant N:P and C:P ratios, especially for the nitrophilous plant species [12]; mowing will suppress the competitive dominance of nitrophilous plant species and facilitate the growth of the entire plant community. Another reason is that long-term N addition might alter ecosystem N status and exacerbate ecosystem phosphorus limitation based on the Stability of Limiting Elements Hypothesis [23].

Regardless of mowing practice, the responses of nutrient stoichiometry to N addition varied among plant components and soils, and the patterns under high-N and low-N inputs were generally not consistent (Figure 1, Figure 2 and Figure 3). In the PCA, colored circles denoting distinct N addition rates were widely separated along the PC1 and PC2 axes, whereas circles for mown versus unmown plots at a given N addition rate were clustered (Figure 3). PC1 alone explained 35.3%, 54.5%, 35.1%, and 50.7% of the total variance in nutrient ratios for aboveground plants, litter, belowground roots, and soils, respectively (Figure 3). Although PC2 alone generally explained less (15–28%) of the total variance as compared with PC1, the distance between low-N addition and high-N addition circles was generally large. Overall, these patterns highlight the importance of N addition rates and suggest that plants tend to sequester more potentially toxic elements (e.g., high Mn concentration) in belowground roots and aboveground litter, while allocating a greater proportion of biomass to aerial tissues, consistent with a dilution effect [49,50]. These results are consistent with the Relative Resorption Hypothesis, which indicated more limited nutrients would be stored in the roots and stems, and other nutrients would be stored in the litter [51]. A recent global meta-analysis reported that N addition increased the plant stem mass fraction by 13.8%, shoot mass fraction by 12.9%, and leaf mass fraction by 13.4%, while decreasing the root–shoot ratio by 27% and root mass fraction by 14.7, respectively [33]. Based on the Productivity-Nutrient Allocation Hypothesis and Stoichiometric Homeostasis Theory, plants will allocate nutrients to different plant organs and maintain the relative stable nutrient stoichiometric at the species level [12,14]. We suspect that these hypotheses and theories are also corrected at the community level. In line with these findings, compared with control plots, high-N addition (10 g m^−2^ year^−1^) significantly reduced the C:N ratio in aboveground plants and litter regardless of mowing (Figure 1a–c), and lowered soil C:N in mown plots. Similar declines were observed in other N-based ratios (N as the numerator), with the exception of the N:Mn ratio in roots and aboveground plants.

Our results also indicate that K, Ca, Mg, Fe, and Mn nutrient ratios merit increased attention in future studies of N fertilization and mowing practices, especially in Haplic Calcisol grassland soils, for the following reasons. First, soil K, Ca, and Mg together account for more than 80% of total base cations in Haplic Calcisol grassland soils; these cations are essential for plant growth and strongly influence the soil acid-neutralizing capacity [36,51,52]. In addition, unlike soil K, Ca, and Mg, which are readily leached into deeper soil layers, plants must absorb substantial amounts of Fe and Mn when soils contain high Fe and Mn concentrations [9,53,54]. Second, given the allocation of elements among different plant organs, mowing removes larger quantities of K, Ca, and Mg than Fe and Mn, and N addition further exacerbates these losses by increasing plant biomass [33,38]. Third, whether a single nutrient limits plant biomass depends not only on its absolute soil content but also on the status of other related nutrients. Thus, analyses of multi-nutrient stoichiometric dynamics can help refine nutrient management strategies. In our experiment, low-N addition (2 g m^−2^ year^−1^) significantly reduced plant P:Mn, K:S, and K:Mn ratios regardless of mowing, whereas high-N addition significantly increased plant P:Fe and P:Mg ratios in unmowed plots and plant P:Fe and K:Fe ratios in mowed plots (Figure 1a,b). Regarding litter nutrient ratios, low-N addition significantly lowered P:Fe and P:K while significantly elevating K:Mn; high-N addition significantly decreased P:K, P:Mg, and P:Mn but increased K:(Ca + Mg) and K:Fe ratios (Figure 1c). In belowground roots and soils, low-N addition significantly reduced the K:S ratio in unmowed plots and P:Mn in mowed plots, whereas high-N addition increased root P:Ca, K:Fe, and K:(Ca + Mg) ratios in both unmowed and mowed plots but decreased P:Mn and K:Mn (Figure 2a,b).

**Figure 4:**
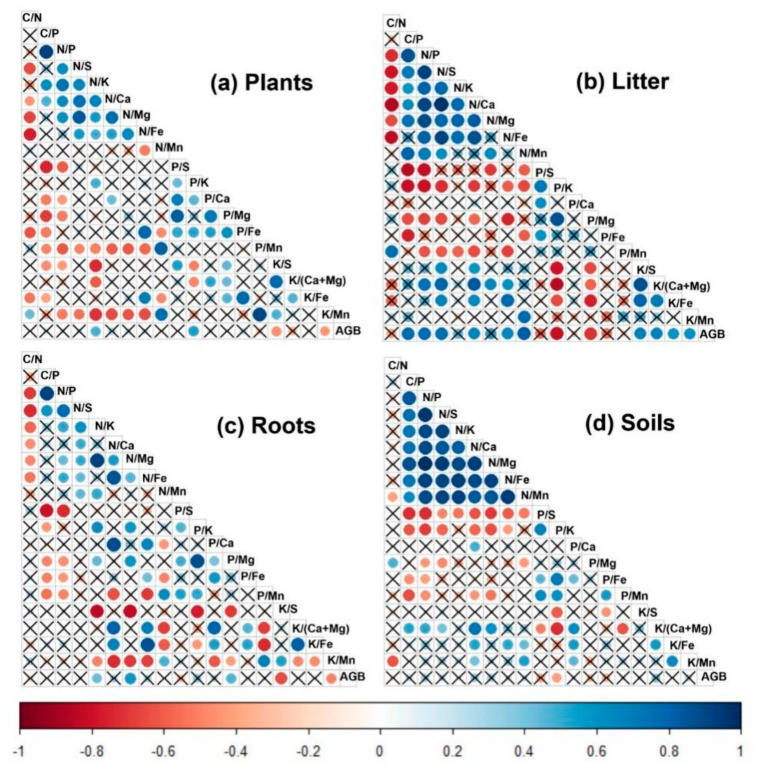
Pearson correlations among 19 nutrient ratios (e.g., C/N represents the C:N ratio) in the plant–soil system. (**a**) Plants; (**b**) litter; (**c**) roots; and (**d**) soils. Notes: the × symbol denotes nonsignificant *Pearson* correlations.

We next examined how nutrient ratios covaried within the plant–soil system and how these ratios were associated with AGB (Figure 4; Table S2). Across plants, litter, and roots, C:N was typically negatively correlated with N-based ratios (N as the numerator), whereas N-related ratios tended to be positively associated with one another within the three plant components and soils (Figure 4a–d). For AGB, we detected significant positive relationships with the N:K and P:K ratios in plants, but significant negative relationships with K:S and K:Mn (*p* < 0.05; Figure 4a,b). In roots, AGB was significantly positively correlated with N:K, N:Mg, and P:Mg, but significantly negatively correlated with K:S and K:Mn; in soils, AGB was negatively correlated with P:K (Figure 4c,d). Together, these findings extend the Productivity-Nutrient Allocation Hypothesis, which emphasizes the key role of different plant organs in mediating the relationship between community biomass and leaf nutrient contents [14]. In addition, we found significant positive correlations between soil C:P and root C:P and between soil N:P and N:P ratio in litter and roots. Likewise, soil N:S was positively related to plant, litter, and root N:S, and soil N:K was positively related to plant and root N:K. Furthermore, soil N:Ca was positively associated with plant and root N:Ca, and soil N:Mg was positively associated with plant and root N:Mg (Table S2). Positive relationships were also detected between soil N:Fe and plant, litter, and root N:Fe, between soil N:Mn and plant N:Mn, between soil P:K and plant and litter P:K, between soil P:Ca and root P:Ca, between soil P:Mn and litter P:Mn, and between soil K:(Ca + Mg) and root K:(Ca + Mg) (Table S2). These cross-compartment stoichiometric relationships among the three plant components and soils indicate that plants can adjust nutrient allocation to better tolerate N addition and mowing, facilitating their maintenance under these management regimes [10,55].

**Figure 5:**
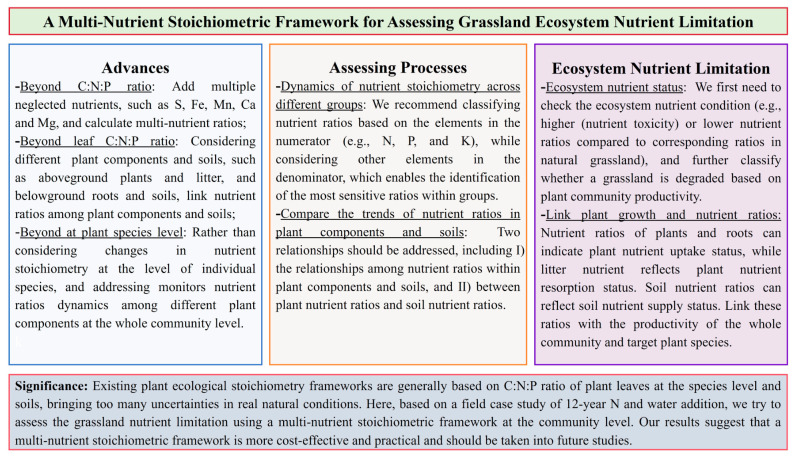
A multi-nutrient stoichiometric framework for assessing nutrient limitation in grassland ecosystems.

We finally proposed a multi-nutrient stoichiometric framework for assessing nutrient limitation in grassland ecosystems (Figure 5), which needs more studies to verify this in grassland ecosystems (also including wet grasslands). The key advantage of this framework is its simultaneous incorporation of multiple stoichiometric ratios—including C:N:P—from different plant components at both the community level and the soil. We recommend classifying nutrient ratios and comparing the trends of nutrient ratios in plant components and soils based on the elements in the numerator (e.g., N, P, and K), while considering other elements in the denominator, and this enables the identification of the most sensitive ratios within groups. Then, we can link plant growth and nutrient ratios, and further assess the ecosystem nutrient limitation. This framework may provide a more feasible and resource-efficient approach to characterizing ecosystem nutrient conditions. However, some limitations in the current study should be clarified and need more research to supplement them. First, given that the present study was conducted using data from only one sampling event at a single site, further validation using repeated measurements from multiple sites is required. Second, we still lack empirical investigations into the relationships between community-level plant nutrient ratios and soil properties across different stages of the growing season (e.g., early and late growing seasons). Species losses induced by N addition and seasonal fluctuations and natural spatial heterogeneity of plants and soils should be treated very carefully. For example, some legume species richness will decline and even go extinct under long-term N addition, while some species (e.g., *Potentilla acaulis* and *Allium tenuissimum*) will disappear before July due to their biological traits in our studied area [48,56]. In addition, the establishment of permanent plots within experimentally manipulated grassland systems, coupled with continuous monitoring, may help overcome the confounding effects of natural spatial heterogeneity. We further suggest using community-level water and carbon-exchange metrics (e.g., evapotranspiration and gross ecosystem photosynthesis) as integrative measures of plant performance. Incorporating stable isotope approaches and other sensitive indicators (e.g., dissolved soil nutrients and exchangeable or available nutrients) may also improve our ability to assess the links between nutrient availability and plant growth [57]. Third, it remains unclear to what extent the nutrient ratios of a subset of dominant plant species can represent the nutrient ratios of the entire plant community at the community level, highlighting an important gap in our understanding of stoichiometric scaling in plant communities.

## 3. Materials and Methods

The experiment was conducted in a typical steppe ecosystem on the Mongolian Plateau in northern China (43°20′ N, 116°40′ E; elevation ~1250 m). The region has a semiarid continental climate and lies near the center of the Eurasian steppe. The soil is classified as Haplic Calcisol, naturally rich in Ca, and the proportions of silt (44.2%) and sand (50.6%) are much greater than that of clay (5.2%) in the 0–10 cm layer [36]. The contents of soil-exchange K, Ca, and Mg are 6.65, 71.35, and 9.61 mmol kg^−1^, respectively; the contents of soil-available Fe and Mn were 0.25 and 0.09 mmol kg^−1^ in 2010 [34]. In September 2008, we established a field experiment on a relatively flat grassland site. The full experimental design was described by Niu et al. (2023) [35]. Here, in brief, there are a total of 10 blocks, and each block contains thirty-eight plots with randomly arranged N addition crossed with two addition frequencies (monthly and twice-yearly) and two levels of mowing (mown and unmown). In the present study, an adjacent four of the 10 blocks were selected randomly to assess the effects of N addition intensity and mowing management on nutrient stoichiometry in aboveground plants and litter, and belowground roots and in the soil. The N addition rates were 0, 2, and 10 g N m^−2^ yr^−1^ with twice-yearly addition under mown (M) and unmown treatments, representing control, low-N, high-N addition, control + M, low-N + M, and high-N + M, respectively. The experimental site has been fenced since 1999 to prevent grazing by large herbivores and had not received any fertilizer before the start of this experiment. Annual autumn mowing was conducted in late August (peak biomass period), and community stubble height was at 10 cm after mowing. Then, we collected plant materials (AGB, litter, and belowground roots) at the community level, together with 0–10 cm soil samples from each plot, in four adjacent blocks between 20 and 25 August 2020. The soil of depth 0–10 cm generally has the highest root biomass and nutrient contents, which are most closely linked to continuous plant growth. It should be noted that we could not collect litter samples in the mown plots because of continuous mowing.

Total C (TC) and total N (TN) in soils were quantified using a C:N elemental analyzer (Analytik-Jena multi N/C3100; Jena, Germany). Total phosphorus (TP), total sulfur (TS), total potassium (TK), total calcium (TCa), total magnesium (TMg), total ferrum (TFe), and total manganese (TMn) in plant tissues and soils were determined after digestion with a strong acid mixture (HClO + HF + HCl), and the resulting solutions were analyzed using an inductively coupled plasma optical emission spectrometer (Thermo Electron Corporation, Waltham, MA, USA). To assess nutrient limitation with a multi-nutrient stoichiometric framework, we calculated 19 nutrient ratios and grouped them into four categories: (i) C as the numerator (C:N; C:P); (ii) N as the numerator (N:P; N:S; N:K; N:Ca; N:Mg; N:Fe; N:Mn); (iii) P as the numerator (P:S; P:K; P:Ca; P:Mg; P:Fe; P:Mn); and (iv) K as the numerator (K:S; K: (Ca + Mg); K:Fe; K:Mn). Further information on materials and methods (e.g., basic soil properties, exchange and available nutrients in mowed and un-mowed plots as shown in Table 1) is provided in the Supplementary Materials.

## 4. Conclusions

Overall, our study showed that long-term N addition resulted in rate-dependent shifts in nutrient stoichiometry, whereas mowing had only weak modifying effects; these responses also differed among the three plant components and the soil. Plant community biomass was associated with nutrient stoichiometric ratios in all three plant components and in soils, although the specific ratios were not the same across compartments. In addition, we characterized stoichiometric linkages among plants, litter, roots, and soils. Given the dynamic nature of nutrient stoichiometry in plant–soil systems, we recommend using a multi-nutrient stoichiometric framework to investigate nutrient limitation (Figure 5), especially through community-level ratios that incorporate often-neglected elements such as Ca, Mg, and Mn.

## Figures and Tables

**Table 1:** Effects of nitrogen (N) addition on the basic soil properties and exchange and available nutrients in mowed and un-mowed plots.

		Control	Low-N	High-N
pH	Unmowed	7.22(0.05) a	7.54(0.28) a	5.75(0.50) b
	Mowed	7.12(0.25)	7.25(0.31)	6.52(0.47)
SOM	Unmowed	35.93(0.87) b	35.76(0.44) b	46.57(3.07) a
	Mowed	37.76(1.94) B	42.70(1.68) AB*	44.26(0.69) A
TN	Unmowed	2.11(0.11) b	2.26(0.06) b	2.59(0.15) a
	Mowed	2.00(0.01) C	2.41(0.08) B	2.72(0.13) A
IN	Unmowed	25.44(0.79) b	29.34(1.24) b	69.74(3.14) a
	Mowed	24.84(0.45) B	25.24(0.51) B	52.10(1.56) A*
Ex.K	Unmowed	310.10(9.84)	260.97(15.38)	235.99(15.82)
	Mowed	302.74(15.43)	260.31(9.18)	251.92(14.29)
EX.Ca	Unmowed	2232.80(51.80) a	2077.04(181.25) a	1629.53(56.26) b
	Mowed	2191.73(58.73) a	2142.96(206.98) a	1963.61(117.30) b*
Ex.Mg	Unmowed	223.20(7.94)	188.84(7.34)	166.34(8.39)
	Mowed	202.23(10.88)	192.29(9.3)	179.71(3.28)
Av.Fe	Unmowed	11.00(0.33) b	8.84(0.40) b	32.20(1.72) a
	Mowed	12.76(1.08) B	11.18(0.64) B	25.58(1.50) A*
Av.Mn	Unmowed	29.58(1.46) b	17.58(1.18) b	78.41(3.17) a
	Mowed	33.48(1.71) B	30.81(1.92) B	69.17(6.53) A

Notes, Data shows as means ± SE (n = 4). Lower-case and capital letters indicate significant differences (*p* < 0.05) between the control and N additional treatments in mowed and un-mowed plots, respectively. Symbols * indicate significance at *p* < 0.05 between mowed and un-mowed treatments at the same N addition rate. Only significant cases were labeled. The unit of all soil factors is mg kg^−1^. The raw data cited Niu et al., 2023 [35]. Soil organic matter, SOM; total N, TN; inorganic N, IN; Exchangeable K, Ca, and Mg, Ex.K, Ex.Ca, and Ex.Mg; Available Fe and Mn, Av.Fe, and Av.Mn.

## Data Availability

The dataset supporting the conclusions of this article is included within the article and can be found in the Supplementary Materials.

## Supplementary Materials

The following supporting information can be downloaded at: https://www.mdpi.com/article/10.3390/plants15142136/s1, Table S1: Pearson correlations between specific nutrient ratios and PC1 axis in plant–soil systems; Table S2: Relationships (linear regression) of soil nutrient ratios with their corresponding nutrient ratios in aboveground plants, litter, and roots.

## Abbreviations

The following abbreviations are used in this manuscript: Aboveground biomass, AGB; Calcium, Ca; Iron, Fe; Magnesium, Mg; Manganese, Mn; Nitrogen, N; Phosphorus, P; Potassium, K.
