# Supplementary material for: A Multi-Nutrient Stoichiometric Framework Reveals Distinct Plant–Soil Responses to 12 Years of Nitrogen Fertilization and Mowing in an Agro-Pastoral Ecotone Grassland
Source: Plants (Basel). 2026 Jul 10;15(14):2136. doi: 10.3390/plants15142136 (PMC13415387; doi:10.3390/plants15142136)
Supplement: Supplementary file 1 [file plants-15-02136-s001.zip › plants-4364032-supplementary.pdf]

Communication

A Multi-Nutrient Stoichiometric Framework Reveals Distinct Plant–Soil Responses to 12 Years of Nitrogen Fertilization and Mowing in an Agro-Pastoral Ecotone Grassland

Muqier Hasi [1,†], Canran Yang [2,†], Yasong Chen [2], Yibo Li [3], Jianhui Huang [4], Yinliu Wang [5] and Guoxiang Niu [2,4,6,*]

[1] College of Grassland Science, Inner Mongolia Agricultural University, Hohhot 010011, China; hasimuqier@imau.edu.cn
[2] Jiangxi Provincial Key Laboratory of Carbon Neutrality and Ecosystem Carbon Sink, Lushan Botanical Garden, Jiangxi Province and Chinese Academy of Sciences, Jiujiang 332900, China; yangcr@lsbg.cn (C.Y.); yasongchen@foxmail.com (Y.C.)
[3] Key Laboratory of Land Surface Pattern and Simulation, Institute of Geographic Sciences and Natural Resources Research, Chinese Academy of Sciences, Beijing 100101, China; liyb.18b@igsnrr.ac.cn
[4] Key Laboratory of Vegetation and Environmental Change, Institute of Botany, Chinese Academy of Sciences, Xiangshan, Beijing 100093, China; jhhuang@ibcas.ac.cn
[5] Shandong Key Laboratory of Eco-Environmental Science for the Yellow River Delta, Shandong University of Aeronautics, Binzhou 256603, China; wangyinliu@ibcas.ac.cn
[6] Senckenberg Museum of Natural History Görlitz, 02826 Görlitz, Germany
* Correspondence: niuguoxiang15@mails.ucas.ac.cn
† These authors contributed equally to this work.

Contents include:

Supplementary Materials and methods; Two tables

1 Supplementary Materials and methods

1.1 Field site and experimental design

The study site is located in a typical steppe (43°20′N, 116°40′E, elevation ~1250 m) in the Mongolia Plateau, northern China. The site has a semiarid continental climate and is central to the Eurasian steppe. The mean annual air temperature is approximately 1.0 °C and yearly precipitation is approximately 320 mm. More than 60% of precipitation events occur from June to August, the main growing season in the study area. The soil type is Haplic Calcisol soil and the contents of silt (44.2%) and sand (50.6%) are remarkably higher than clay content (5.2%) in the 0-10 cm soils (Niu et al., 2021). Five perennial grasses, such as *A. cristatum, A. sibiricum, Artemisia scoparia Waldst, L. chinensis, and S. grandis,* dominated the plant community, and their total biomass comprised more than 85% of the community's biomass according to the plant community surve during 2018-2020.

In September 2008, we selected a relatively flat grassland to set up our field experiment. The study area was fenced to exclude feeding from cattle and sheep in 1999. The 10 blocks were established using a completely randomized block design, and each block contains thirty-eight plots with randomly arranging 9 levels of N addition crossed with two addition frequencies (monthly and half-yearly) and two levels of mowing (mown and unmown). The detailed experiment was described by Niu et al. (2021). Here, only three N addition rates (0, 2, and 10 g N m^{-2} yr^{-1} with half-yearly addition under mown (M) and unmown treatments, representing control, low-N, high-N addition, control+M, low-N+M, and high-N+M, respectively) in 4 out of 10 blocks were selected to assess the effects of N addition intensity and mowing management on nutrient stoichiometry in aboveground plants, litter, and belowground roots and in the soil. Since 2009, NH_4NO_3 has been uniformly applied in equal amounts on the first day of November and June, and mowing has been performed in late August by retaining plant stubble height of 10 cm.

1.2 Plant material and soil sampling

After 12 years of consecutive N addition of 2 and 10 grams per year, we sequentially collected plant material (aboveground plants, litter, and belowground roots) at the community level and soil samples from each plot in the adjacent 4 blocks on 20-25th August 2020 (Niu et al., 2023). Specifically, the green above-ground plants from three randomly 0.25 m × 0.25 m quadrats in each subplot within 4 blocks, and the litter was collected by hand. Due to the green plant having been removed in the previous year, litter was not collected in the mown plots. Surface (0-10 cm) soil and root samples were collected using a

corner and the mixed samples were further sieved with a 2-mm mesh to separate soil or root samples. All soil and plant (three parts of plants) samples from three quadrats were fully mixed, and 71 plant material samples (24 for aboveground plants, 23 for litter, and 24 for belowground roots) and 24 soil samples were collected. Samples of plants, litter, and roots were dried in an oven, while soil samples were air-dried. Both two types of samples were ground for further analysis.

1.3 Measurements related to plant and soil samples

The content of total C (TC) and total N (TN) in all soils was determined with a C/N elemental analyzer (Analytik-Jena multi N/C3100; Jena, Germany). Contents of total phosphorus (TP), total sulfur (TS), total potassium (TK), total calcium (TCa), total magnesium (Mg), total ferrum (TFe), and total manganese (TMn) in plant materials and soils digested with the strong acid mixture (HClO + HF + HCl) and the digested liquid was measured with an inductively coupled plasma optical emission spectrometer (Thermo Electron Corporation, Waltham, Massachusetts, USA). Specifically, 0.50 g of ground soil samples and 1.00 g of ground plant materials were weighed and put into sealed polytetrafluoroethylene jars with the high-pressure reactor outside. The 2 mL of HClO, 2 mL of HF, and 8 mL of HCl were sequentially added into the jar and covered with the high-pressure reactor. The whole high-pressure reactor was translocated in an oven at 150 °C for at least 4 h until plant materials or soil samples were fully dissolved in the mixed strong acid. After removing excess acid from polytetrafluoroethylene jars under a lower temperature (less than 80 °C), the residual solids were dissolved using 5% HNO_3 and further measured with the optical emission spectrometer.

1.4 Statistics

We performed the statistical processes in R4.1.2, R3.6.2, and R studio. First, we uniform the units of nine element contents. To better explore nutrient limitations using multi-nutrient stoichiometry, we calculated 19 nutrient rations and further divided these ratios into four categories: i) C as the numerator (C:N; C:P); ii) N as the numerator (N:P; N:S; N:K; N:Ca; N:Mg; N:Fe; N:Mn); iii) P as the numerator (P:S; P:K; P:Ca; P:Mg; P:Fe; P:Mn); iv) K as the numerator (K:S; K: Ca+Mg; K:Fe; K:Mn). After testing the homogeneity and normality of our data, we used a two-way analysis of variance (ANOVA) to show the main effects of N addition and mowing on the changes in nutrient stoichiometry in three plant components and soils. Following the step, we used a one-way ANOVA to show the effects of N addition rates in mown and unmown plots and the differences between mown and control plots. Furthermore, we used principal component analysis with permutational multivariate analysis of variance to show the

overall effects of N addition and mowing on 19 nutrient rations. We used Pearson correlation analyses to identify better the correlations between these rations in aboveground plants, litter, belowground roots, and soils. Following Pearson correlation analyses, we used linear regression analyses to show the relationships between soils and three plant components.

2 Supplementary Tables

Table S1 Pearson correlations between specific nutrient ratios and PC1 axis in plant-soil systems. ***, **, and * indicate significant correlations at $p < 0.001$, 0.01, and 0.05, respectively.

	**Plants**	**Litter**	**Roots**	**Soils**
**C:N**	-0.68***	-0.71**	-0.71**	-0.28
**C:P**	0.41*	0.83**	0.83**	0.81***
**N:P**	0.67***	0.97***	0.97***	0.98***
**N:S**	0.79***	0.95***	0.95**	0.92***
**N:K**	0.79***	0.71**	0.71*	0.89***
**N:Ca**	0.81***	0.93***	0.93***	0.96***
**N:Mg**	0.84***	0.81***	0.81***	0.97***
**N:Fe**	0.82***	0.85	0.85***	0.95***
**N:Mn**	-0.56**	0.69	0.69	0.90***
**P:S**	-0.03	-0.72	-0.72*	-0.71***
**P:K**	0.30	-0.88	-0.88	-0.65***
**P:Ca**	0.18	-0.05	-0.05***	0.25
**P:Mg**	0.13	-0.77	-0.76	-0.38
**P:Fe**	0.47*	-0.43	-0.43	-0.28
**P:Mn**	-0.81***	-0.62	-0.62***	-0.46
**K:S**	-0.37	0.66	0.66	0.08
**K:(Ca+Mg)**	-0.16	0.84	0.84***	0.62***
**K:Fe**	0.29	0.66	0.66***	0.51*
**K:Mn**	-0.89***	0.27	0.27***	0.40

Table S2 Relationships (linear regression) of soil nutrient ratios with their corresponding nutrient ratios in aboveground plants, litter, and roots. ***, **, and * indicate significant correlations at $p < 0.001$, 0.01, and 0.05, respectively.

Soil	Plants	Litter	Roots
**C:N**	**-0.05**	**0.01**	**-0.04**
**C:P**	**0.01**	**-0.03**	**0.14***
**N:P**	**-0.1**	**0.33***	**0.14***
**N:S**	**0.34***	**0.28***	**0.17***
**N:K**	**0.15***	**0.13**	**0.15***
**N:Ca**	**0.40***	**0.21**	**0.77***
**N:Mg**	**0.38***	**-0.05**	**0.19***
**N:Fe**	**0.59***	**0.51***	**0.56***
**N:Mn**	**0.18***	**0.04**	**-0.03**
**P:S**	**0.11**	**0.1**	**-0.04**
**P:K**	**0.14***	**0.28***	**-0.04**
**P:Ca**	**-0.01**	**-0.1**	**0.17***
**P:Mg**	**-0.01**	**0.05**	**-0.04**
**P:Fe**	**-0.04**	**-0.1**	**0.02**
**P:Mn**	**-0.04**	**0.26***	**0.07**
**K:S**	**-0.04**	**-0.03**	**-0.04**
**K:(Ca+Mg)**	**-0.05**	**0.07**	**0.47***
**K:Fe**	**-0.01**	**0.08**	**0.07**
**K:Mn**	**-0.02**	**0.04**	**-0.03**
